# Suicide risk in high school students: who are the most vulnerable groups?

**DOI:** 10.1590/1984-0462/2023/41/2021236

**Published:** 2022-07-06

**Authors:** Laura Silva da Silva, Priscila Arruda da Silva, Lauro Miranda Demenech, Maria Eduarda Centena Duarte Vieira, Lucas Neiva Silva, Samuel Carvalho Dumith

**Affiliations:** a Universidade Federal do Rio Grande, Rio Grande, RS, Brazil.; b Universidade Católica de Pelotas, Pelotas, RS, Brazil.

**Keywords:** Suicide, Health risk behavior, Adolescents, Cross-sectional studies, Mental health, Suicídio, Comportamentos de risco à saúde, Adolescentes, Estudos transversais, Saúde mental

## Abstract

**Objective:**

To investigate the prevalence and factors associated with suicide risk among high school students from a federal educational institution in Rio Grande do Sul (IFRS).

**Methods:**

This is a cross-sectional study based on a census of students (n=510) enrolled in IFRS, campus Rio Grande, in the second half of 2019. Data were collected through a self-administered questionnaire. Suicide risk was measured with the instrument Mini-International Neuropsychiatric Interview, and data were analyzed using Poisson regression with robust variance adjustment.

**Results:**

The prevalence of high suicide risk was 17.3% (95% confidence interval — 95%CI 14.0–20.0), with the following independent associated factors: female gender, higher socioeconomic status, alcohol consumption, less social support, attempt to lose weight, self-harm behavior, and increased risk of depression, anxiety, and stress.

**Conclusions:**

One in six students showed a high suicide risk. The identification of factors associated with the outcome is useful for detecting the most severe cases and referring them to specialized care.

## RESUMO

**Objetivo:**

Investigar a prevalência e os fatores associados ao risco de suicídio em estudantes do ensino médio de uma instituição federal de ensino do Rio Grande do Sul (IFRS).

**Métodos:**

Trata-se de um estudo transversal realizado com base em um censo de estudantes (n=510) matriculados no IFRS, *Campus* Rio Grande, no segundo semestre de 2019. Os dados foram coletados por meio de questionário autoaplicável. O risco de suicídio foi medido com o instrumento *Mini-International Neuropsychiatric Interview* e a análise dos dados foi conduzida por meio de regressão de Poisson com ajuste robusto da variância.

**Resultados:**

A prevalência do risco alto de suicídio foi de 17,3% (intervalo de confiança — IC95% 14,0–20,0), tendo como fatores independentemente associados: sexo feminino, maior nível socioeconômico, consumo de álcool, menor suporte social, tentativa de perder peso, comportamento autolesivo e maior risco de depressão, ansiedade e estresse.

**Conclusões:**

Um em cada seis estudantes apresentou elevado risco de suicídio. A identificação dos fatores associados ao desfecho é útil para identificar os casos mais graves e encaminhá-los para atendimento especializado.

## INTRODUCTION

In adolescence, young people tend to experience great changes, acquire new skills, and face challenges, which can trigger suicidal behaviors.^1^ In recent decades, suicidal behavior has grown among young students, with a strong association with contemporary lifestyles centered on solitude, digital distractions, individualism, exacerbated competitiveness, and lack of public policies.^2^

Suicide is among the top 20 causes of death for all ages worldwide. Every 40 seconds, one person commits suicide in the world.^3^ Brazil is among the ten countries with the highest absolute numbers of suicide. From 1996 to 2015, 172,051 deaths were recorded as suicide, with a progressive increase in all country regions.^4^

Study carried out in 90 countries^5^ shows a high prevalence of suicide rates among young people aged 15 to 19 years, with 46.5% in Sri Lanka, 23.9% in Lithuania, and 23.6% in Russia. Canada held the 15^th^ position (10.8%) and the United States, the 34^th^ position (8%). In Brazil, the estimate was 4.2%; in large Brazilian cities, the suicide rate among adolescents increased 24% between 2006 and 2015.^6^

Among adolescents, the factors that increased suicide risk were: poverty, violence, economic differences, family conflict, use of psychoactive substances, less social support, disappointment in love, solitude, family history, low school performance.^7–9^

The mental health of students has been a recurrent theme in research. However, despite the significant suicide rates in this population, adolescents have not received the necessary attention from government agencies responsible for promoting public policies. Faced with the demands of school life and adolescence, these students may be exposed to stress agents, leading to illnesses.^9^

Given the increasing number of deaths by suicide in Brazil and worldwide and the gap in information about suicide risk among young students, knowing this public health problem better becomes necessary. Thus, the overall objective of the present study was to estimate the prevalence of suicide risk and investigate the factors associated with this risk in high school students from a federal educational institution in Rio Grande do Sul (IFRS).

## METHOD

This is a population-based cross-sectional study carried out in an IFRS located in the far south of Rio Grande do Sul.^10^ All students attending the vocational high school at IFRS (campus Rio Grande) in the second half of 2019 were included in this research. A total of 718 students were enrolled from the 1^st^ to the 4^th^ grade of vocational high school, comprising the target population of the study.

After approval from the Research Ethics Committee and authorization from the educational institution, first contact was made with the classes to present the research and deliver the informed consent form. Students aged 18 and older signed the form, and those under the age of 18 asked their parents to sign. Students who agreed to participate received the self-administered and anonymous questionnaire on paper. Those who chose not to participate in the research or were not found after three visits to the classroom were considered losses. In the administration of the questionnaire, we verified whether all enrolled students answered the survey. This verification consisted of comparing the total number of enrollments with the total number of students present (respondents and refusals). After identifying the students’ absence in class, the supervisor scheduled a new visit to find these students. Data were collected for two weeks, from Monday to Friday, in three periods (morning/afternoon/evening). At the end of data collection, the students dropped the questionnaires in a collection box.

The dependent variable of this study was suicide risk, obtained by the instrument Mini-International Neuropsychiatric Interview,^11^ adapted for self-administration. Responses to this scale produced a score categorized as follows: no risk (0 points), low risk (1 to 5 points), moderate risk (6 to 9 points), and high risk (10 points or more).^11^ This study analyzed the group with high suicide risk as the outcome.

The independent variables included in this research were: gender (male or female); age (divided into age groups: 14–15, 16–17, 18–20); ethnicity (white or other); maternal schooling (elementary school, high school, or higher education); goods index (built by analyzing main components, divided into quartiles from lowest to highest); leisure-time physical activity (no or yes); tobacco use (no or yes); alcohol consumption in the previous month (no or yes); excessive use of social media (5 hours or more), assessed by the question “How long do you spend on social media (Facebook, Instagram, Snapchat, or other)?”; overweight according to the World Health Organization (WHO) criterion — presenting a Z score above 1.00 based on growth curves,^12^ with self-reported weight and height; social support^13^ (score divided into quartiles from highest to lowest); attempt to lose weight (no or yes); being bullied at school (no or yes); self-harm behavior in the previous year (no or yes), assessed by the question “Have you ever hurt yourself on purpose (for example: have you cut, burned, scratched, pierced, hit, or rubbed yourself excessively) without the intention of killing yourself?”; symptoms of depression, anxiety, and stress^14^ (score divided into quartiles from lowest to highest).

The data obtained from the self-administered questionnaires were entered into the EpiData 3.1 software and subsequently transferred to and analyzed in the Stata 15.1 statistical package. The sample was described according to independent variables, using absolute and relative frequency. Next, we conducted a bivariate analysis, which showed the prevalence of high suicide risk according to independent variables.

Lastly, Poisson regression with robust variance adjustment was performed to run the crude and adjusted analyses, extracting the prevalence ratio (PR), 95% confidence interval (95%CI), and p-value from the Wald test. The adjusted analysis used a four-level hierarchical analysis model, in which each variable was controlled for those of the same and higher levels. Variables were selected using the backward method. The significance level adopted was 5% for two-tailed tests.

The Research Ethics Committee of Universidade Federal do Rio Grande (FURG) approved this research project under opinion no. 128/2018, Certificate of Presentation for Ethical Consideration (*Certificado de Apresentação para Apreciação Ética* — CAAE) no. 91281918.7.0000.5324. Students were informed about the voluntary participation in the research and the possibility of not answering the questionnaire or taking action at any time. They were also aware that if any health need was identified during the interviews or a positive result was obtained for some disease, referral to the following services would be offered: Health Care Center (*Núcleo de Atenção à Saúde* — NAS) of the facility and FURG's psychology service.

## RESULTS

Among the 718 students enrolled, 84 had dropped the course at the time of data collection, totaling 634 students eligible to compose the sample. Of them, 25 refused to participate, and 92 were not found, resulting in an 81.5% response rate. The dependent variable of this study had seven missing data, which led to a final sample size of 510 individuals. The prevalence of high suicide risk was 17.3 (95%CI 14.0–20.6).

The participants’ ages ranged from 14 to 20 years; most were white individuals (77.1%), with no gender predominance. Three-quarters of the sample did not practice physical activity, 28.4% reported having attempted to lose weight, and 30.5% were overweight. The maternal schooling of 36.8% of the participants was complete high school. Half of the sample reported having consumed alcohol at least once, and 6.5% have used tobacco products at least once. The prevalence of excessive use of social media was 36.4%. One-third (35.3%) declared having been bullied, and 18.8% showed self-harm behavior (Table 1).

**Table 1:** Description of the sample of high school students from Rio Grande (RS), Brazil, 2019 (n=510).

		n	%
Gender			
	Male	257	50.6
	Female	251	49.4
Age (years)			
	14–15	98	19.4
	16–17	247	48.8
	18–20	161	31.8
Ethnicity			
	White	390	77.1
	Other	116	22.9
Maternal schooling			
	Elementary school	66	13.7
	High school	178	36.8
	Higher education	239	34.5
Goods index			
	1^st^, 2^nd^, and 3^rd^ quartile	379	75.3
	4^th^ quartile (richer)	124	24.7
Physical activity			
	No	394	79.3
	Yes	103	20.7
Tobacco use			
	No	473	93.5
	Yes	33	6.5
Alcohol consumption			
	No	259	51.0
	Yes	249	49.0
Excessive use of social media			
	No	322	63.6
	Yes	184	36.4
Overweight			
	No	346	69.5
	Yes	152	30.5
Social support			
	1^st^, 2^nd^, and 3^rd^ quartile	382	76.4
	4^th^ quartile (less support)	118	23.6
Attempt to lose weight			
	No	365	71.6
	Yes	145	28.4
Bullying			
	No	330	64.7
	Yes	180	35.3
Self-harm behavior			
	No	414	81.2
	Yes	96	18.8
Depression, anxiety, stress			
	Lower risk	394	80.1
	Higher risk	98	19.9

Table 2 presents the results of the prevalence of suicide risk according to the independent variables of the study, as well as the findings of the crude and adjusted analyses. This table shows that high suicide risk was more prevalent among individuals with increased risk of depression, anxiety, and stress (54.1%), self-harm behavior (46.9%), tobacco use (42.4%), and lower social support scores (38.1%). The probability of high suicide risk was lower among participants who reported not being bullied (12.7%), did not consume alcohol (11.6%), were male (11.3%), and practiced physical activity (10.7%).

**Table 2:** Crude and adjusted analysis of factors associated with high suicide risk among high school students from Rio Grande (RS), Brazil, 2019 (n=510).

Level			Suicide risk (%)	Crude analysis		Adjusted analysis	
				PR (95%CI)	p-value	PR (95%CI)	p-value
1	Gender						
		Male	11.3	1.00	0.001	1.00	<0.001
		Female	23.1	2.05 (1.36–3.09)		2.20 (1.45–3.35)	
1	Age (years)						
		14–15	13.3	1.00	0.244	1.00	0.226
		16–17	19.8	1.50 (0.85–2.63)		1.72 (0.92–3.22)	
		18–20	14.9	1.12 (0.60–2.10)		1.46 (0.73–2.90)	
1	Ethnicity						
		White	15.1	1.00	0.037	1.00	0.042
		Other	23.3	1.54 (1.03–2.31)		1.52 (1.02–2.28)	
1	Maternal schooling						
		Elementary school	13.6	1.00	0.576	1.00	0.655
		High school	18.5	1.36 (0.69–2.69)		1.30 (0.69–2.48)	
		Higher education	15.5	1.14 (0.58–2.23)		1.11 (0.59–2.11)	
1	Goods index						
		1^st^, 2^nd^, and 3^rd^ quartile	14.8	1.00	0.041	1.00	0.012
		4^th^ quartile (richer)	22.6	1.53 (1.02–2.29)		1.67 (1.12–2.48)	
2	Physical activity						
		No	19.0	1.00	0.053	1.00	0.199
		Yes	10.7	0.55 (0.31–1.01)		0.68 (0.38–1.23)	
2	Tobacco use						
		No	15.6	1.00	<0.001	1.00	0.063
		Yes	42.4	2.71 (1.73–4.25)		1.64 (0.97–2.77)	
2	Alcohol consumption						
		No	11.6	1.00	0.001	1.00	0.039
		Yes	23.3	2.01 (1.34–3.02)		1.57 (1.02–2.42)	
2	Excessive use of social media						
		No	13.4	1.00	0.003	1.00	0.066
		Yes	23.9	1.79 (1.23–2.62)		1.45 (0.98–2.15)	
3	Overweight						
		No	16.2	1.00	0.538	1.00	0.957
		Yes	18.4	1.14 (0.75–1.72)		0.99 (0.67–1.46)	
3	Social support						
		1^st^, 2^nd^, and 3^rd^ quartile	10.7	1.00	<0.001	1.00	<0.001
		4^th^ quartile (less support)	38.1	3.55 (2.46–5.14)		3.45 (2.28–5.21)	
3	Attempt to lose weight						
		No	13.7	1.00	0.001	1.00	0.004
		Yes	26.2	1.91 (1.31–2.79)		1.86 (1.22–2.82)	
4	Bullying						
		No	12.7	1.00	<0.001	1.00	0.931
		Yes	25.6	2.01 (1.38–2.93)		1.02 (0.71–1.45)	
4	Self-harm behavior						
		No	10.4	1.00	<0.001	1.00	<0.001
		Yes	46.9	4.51 (3.17–6.43)		2.20 (1.50–3.24)	
	Depression, anxiety, stress						
		Lower risk	7.6	1.00	<0.001	1.00	<0.001
		Higher risk	54.1	7.10 (4.81–10.5)		3.47 (2.11–5.71)	

PR: prevalence ratio; 95%CI: 95% confidence interval.

After adjustments, the following factors were identified as independently associated with suicide risk: being female (PR 2.20; 95%CI 1.45–3.35), belonging to the highest goods index quartile (PR 1.67; 95%CI 1.12–2.48), having consumed alcohol in the previous month (PR 1.57; 95%CI 1.02–2.42), having less social support (PR 3.45; 95%CI 2.28–5.21), attempting to lose weight (PR 1.86; 95%CI 1.22–2.82), showing self-harm behavior (PR 2.20; 95%CI 1.50–3.24), and having a higher level of depression, anxiety, and stress (PR 3.47; 95%CI 2.11–5.71) (Table 2). The final analysis model explained 24% of the outcome variability.

## DISCUSSION

This article presents results on suicide risk among adolescent students, allowing us to identify groups at higher risk. The prevalence of high suicide risk in this study (17.3%) is similar to values found in Chile (20.1%)^9^ and Colombia (16.5%).^15^ A study carried out in Southern Brazil with adolescent students identified that one in ten students presented a high suicide risk, while in the present study, the proportion found was almost twice that number: one in every six.^16^ According to WHO, between 2010 and 2016, the trend in suicides increased 6% in the American continent.^3^ In Brazil, suicide rates grew 47% among young people aged 15 to 29 years from 2000 to 2015.^5^ These results show that the increased prevalence of high suicide risk detected in this research follows epidemiological estimates.

Girls presented twice the probability of suicide risk compared to boys, an association consistent with the scientific literature.^17,18^ In a study on the prevalence and associated factors in adolescent students from the metropolitan area of Porto Alegre,^19^ the prevalence of suicide risk among female adolescents was 6.3%; in the present investigation, this prevalence was four times higher (23.1%). Regarding associated factors, adolescents who reported feeling lonely and sad showed a higher prevalence of suicidal ideation than those without these feelings. The higher suicide risk among girls can be explained by the association of greater exposure to the main behaviors with other risk factors, such as suicidal ideation, previous suicide attempt, depression, anxiety, and stress. In addition, the stronger social pressure on the female body, a consequence of a sexist culture, may act as a risk factor for mental health.^20^ We underline that, despite the lower exposure to suicide risk factors, boys have a higher suicide rate and use more aggressive methods.^20^

Students with more goods had a higher suicide risk. This result was not expected since lower socioeconomic status tends to have a greater association with suicide in the scientific literature.^7,5,17^ Therefore, the higher suicide risk among students with a better socioeconomic status may be a peculiarity of our target population, and other studies are necessary to confirm or refute this finding. Individuals with higher socioeconomic status might suffer greater social pressure for academic and professional success, a factor that has been associated with increased anxiety and depression symptoms among adolescents.^21^

This hypothesis is in line with another finding of this study: students with less social support from family and friends had a three-fold higher suicide risk. Family dysfunction and the lack of social support and connection between family members have been associated with suicidal behavior.^22^ Adolescence is a period of biopsychosocial transformations in which the individual experiences significant changes in their development; thus, family support is crucial to help with emotional demands and encourage healthy lifestyles.^7^ In addition, identification with the social group is relevant because it provides a sense of belonging and the construction of emotional support networks, with the interaction between adolescents and their peers being essential.^7^ As a result, students with less social support may feel more helpless when dealing with their emotional difficulties and demands, which contributes to the increase in suicide risk.

In this study, suicide risk was 73% higher among participants who reported having attempted to lose weight — not those who were overweight. This result points to the risk of a mistaken perception of weight and body image associated with psychological suffering. For instance, a study identified that almost 40% of the association between obesity and depression relates to the perception of being fat^23^ Students who attempt to lose weight are likely those who are more dissatisfied with their bodies, leading to low self-esteem and, consequently, a higher probability of depressive symptoms and suicidal ideation.^24^

Alcohol consumption in the previous month was a factor connected to high suicide risk. Adolescents with early use of substances, especially alcohol, present a higher suicide risk. Alcohol consumption among adolescents is associated with greater psychological suffering, particularly due to anxiety, depression, and stress.^16,18^ In this context, alcohol may be used as a coping mechanism to deal with difficult emotions, contributing to the distraction from/avoidance of problems.^25^ Alcohol might also have a physiological effect on adolescents, as this substance may induce and/or increase depressive thoughts and feelings.^26^

The results of the present research are in line with the scientific literature since individuals with self-harm behavior had twice the probability of suicide risk. Self-harm behaviors are acts that the person commits with the intention of hurting themselves but not necessarily of causing their own death.^27^ Cutting the wrists, for example, can be a way of relieving psychological pain, replacing it with physical pain. They are very frequent in adolescence and are associated with psychiatric diseases, such as anxiety disorders, depressive disorders, and high levels of stress, and with a significant increase in suicide risk. Data from international studies show that around 10% of young people have experienced at least one episode of self-harm at some point in their lives.^28^ Individuals with self-harm behavior might present a greater level of psychological suffering, which contributes to them thinking of, planning, and even attempting to commit suicide.

Participants with stronger symptoms of depression, anxiety, and stress were more likely to be at risk of committing suicide. According to a systematic review of longitudinal studies on suicide, mental disorders (especially mood disorders and anxiety disorders) and other psychological comorbidities increased the risk for suicide attempts among young people.^29^ This association reflects the notion that individuals with emotional difficulties are at greater risk of considering suicide as a feasible alternative to interrupt their suffering.

Lastly, the results of this investigation should be interpreted taking into account its limitations. First, the cross-sectional design does not allow us to establish temporality and may lead to reverse causality bias (especially in associations between suicide risk and behavioral variables). Second, the prevalence of suicide risk may have been underestimated because students who did not want to participate or did not attend classes may be at higher suicide risk. Despite the many measures taken to ensure secrecy and confidentiality, given the personal and emotionally difficult nature of these questions, some participants may have provided false answers to avoid further exposure. Moreover, this study was carried out before the COVID-19 pandemic, and the high prevalence of suicide risk reported herein might be even greater in the current scenario. Unpublished data from the replicate of this study with a sample of students from the same institution collected in 2020 show that fear of the pandemic increased alcohol consumption, which was a risk factor for suicidal behavior.

Based on this study, we can conclude that one in six students from a high school in Southern Brazil presented high suicide risk. This outcome was independently associated with the female gender, higher socioeconomic status, alcohol consumption in the previous month, and less support from family and friends. Some measures may be adopted based on these results. The themes “psychological suffering” and “suicide” must be included in academic activities beyond one-off events (such as lectures). Schools can create and/or strengthen devices capable of proposing activities for prevention and promotion of mental health, as well as identifying severe cases, helping them, and, when necessary, referring them to specialized monitoring. This screening should take into account the associated factors detected in this research since young people with these characteristics might belong to the most vulnerable groups.
